# Supplementary material for: Non-zero mean alpha oscillations revealed with computational model and empirical data
Source: PLoS Comput Biol. 2022 Jul 8;18(7):e1010272. doi: 10.1371/journal.pcbi.1010272 (PMC9269450; doi:10.1371/journal.pcbi.1010272)
Supplement: S1 Appendix — In the following appendix, we provide additional simulations to show that the estimation of BSI is not biased when the oscillatory signal has a 1/f spectrum. (PDF) [file pcbi.1010272.s001.pdf]

S1 Appendix
BSI performance on simulated data with 1/f noise

The quantification of baseline shifts via baseline-shift index (BSI) [1] includes bandpass-filtered signal and low-frequency signal. It is now acknowledged and a widely researched matter that electrophysiological data are characterised by spectra with 1/f frequency distribution. Therefore, neuronal processes with a 1/f spectrum contribute to the signal in a broad frequency range and may thus affect the estimation of BSI. To test this, we performed additional simulations.

Methods

Firstly, we tested whether BSI would detect non-zero mean oscillations in a case when oscillations are absent. We simulated 900 time series of a 6-min-long signal consisting of either 1/f noise or a combination of 1/f noise and white noise (pink noise was simulated with python module colorednoise which is based on [2]). However, the actual null hypothesis for BSI is that alpha oscillations have a zero mean (see eq. 1, main text). Therefore, secondly, we simulated 900 time series of a 6-min-long signal consisting of 1/f noise, and zero-mean or non-zero mean alpha oscillations. We simulated amplitude-varying alpha oscillation as a white noise filtered in the 8-12 Hz [3, 4]. To construct non-zero mean oscillations, we summed oscillations with their rectified copy multiplied by a factor of 0.4. This situation scenario mimics exactly the eq. 1. For each time series, we computed BSI in the following way. From a composite time course, we obtained amplitude envelope (V_{alpha}) by applying the Hilbert transform to a band-pass filtered signal in the 8-12 Hz range and baseline shifts (V_{bs}) by low-pass filtering the signal at 3 Hz. After, V_{alpha} was divided into 20 percentile bins according to its magnitude. V_{bs} were sorted using the arrangement of the bins from V_{alpha}. The relation between V_{alpha} and V_{bs}, which is, in fact, a BSI, was estimated with the Pearson correlation coefficient (see also section Methods/The baseline-shift index). Each BSI was subjected to permutation testing to determine its significance (see section Methods/Statistical analysis).

Results

Signals that contained only noise did not show evidence for non-zero mean oscillations. For both 1/f noise or 1/f and white noise, the average BSI magnitude was 0.37. However, the fraction of BSIs that were significantly different from zero based on permutation testing was at a chance level of 0.05. The null hypothesis that oscillations have a zero mean and the alternative hypothesis of non-zero mean oscillations were successfully evaluated with BSI as well. The average value of BSI was 0.36 for zero-mean oscillations and 0.85 for non-zero mean oscillations (at an average signal-to-noise ratio in the alpha band of 9 dB). The share of BSIs that were significantly different from zero based on permutation testing was 0.84 for non-zero mean oscillations. The share of non-zero BSIs for zero-mean oscillations was at the significance level.

References

1. Nikulin VV, Linkenkaer-Hansen K, Nolte G, Curio G. Non-zero mean and asymmetry of neuronal oscillations have different implications for evoked responses. Clinical Neurophysiology. 2010;121(2):186–193.

2. Timmer J, Koenig M. On generating power law noise. Astronomy and Astrophysics. 1995;300:707.

3. Nikulin VV, Nolte G, Curio G. A novel method for reliable and fast extraction of neuronal EEG/MEG oscillations on the basis of spatio-spectral decomposition. NeuroImage. 2011;55(4):1528–1535.

4. Idaji MJ, Müller KR, Nolte G, Maess B, Villringer A, Nikulin VV. Nonlinear interaction decomposition (NID): A method for separation of cross-frequency coupled sources in human brain. NeuroImage. 2020;211:116599.
