# Supplementary material for: Non-zero mean alpha oscillations revealed with computational model and empirical data
Source: PLoS Comput Biol. 2022 Jul 8;18(7):e1010272. doi: 10.1371/journal.pcbi.1010272 (PMC9269450; doi:10.1371/journal.pcbi.1010272)
Supplement: Attachment — Submitted filename: response_to_reviewers.pdf [file pcbi.1010272.s002.pdf]

Dear editors,

We carefully examined reviewers' comments and suggestions and edited our manuscript accordingly. We are grateful to reviewers as they provided a kind attitude paired with constructive feedback. The edited version of the manuscript, we believe, is clearer and more digestible for the reader. Below please find our answers to the comments. The revised manuscript with and without edits is attached in a separate file.

Reviewer #1: *In this paper Studenova et al investigate whether neural oscillations and evoked potentials could (at least in some cases) reflect the same underlying phenomena. In particular, they investigate the idea that neural oscillations may have a non-zero mean, and that this, paired with amplitude modulation in response to stimuli, can plausibly explain evoked potentials. To investigate this, they use both simulations, using the Human Neocortical Neurosolver tool, and empirical data analysis, measuring the baseline-shift index, a putative marker of non-zero mean oscillations, in EEG data from young and old participants. They report that both the modelling and empirical analyses are consistent with non-zero mean oscillations, in support of the baseline-shift mechanism of evoked potentials.*
Overall, I find this paper to be an interesting and useful contribution to conceptualizing and investigating features of neural data, and I think the main analyses are sensible and compelling. I particularly like the combination of simulation and empirical work, and in particular the use of modelling that addresses the potential underlying physiology. Overall, I think this paper makes a useful contribution to the literature. I have some relatively minor comments and suggestions for the manuscript, in terms of a question about the methods and some notes on the discussion of the findings.
I feel that, at times, the paper and results don't clearly differentiate if the findings support the potential contribution of the baseline-shift mechanism (with other potential mechanisms still in play) or whether they suggest that baseline-shifts ubiquitously explain evoked potentials. For example, the final sentence of the abstract states that "Overall, our results provide strong support for the unification of neuronal oscillations and evoked responses." Where I find the paper convincing in arguing that there exist non-zero mean oscillations, and that these plausibly at least contribute to evoked-potentials, to claim there is a convincing demonstration that the oscillations and evoked potentials can be completely merged, as is implied here, is not demonstrated. The paper itself acknowledges this in other places, stating "It should be noted that these mechanisms are not assumed to be mutually exclusive and may co-exist" in the introduction. I think the abstract should be careful not to overstate the overall conclusion, and this line should be edited.

We absolutely agree with this comment. We admit that partially our statements were overenthusiastic. We rewrote the abstract in line with this comment and comments

from another reviewer (see later). Additionally, we edited several sentences within the Introduction and Discussion section that contained exaggerated claims.

In the Introduction, 1st paragraph: “On the other hand, evoked responses may, in fact, reflect changes in certain aspects of neuronal oscillations from which it would follow that both phenomena may be closely linked and share a neurophysiological origin.”

In the Introduction, 5th paragraph: “The intuition about oscillations having non-zero OM stems from asymmetrical morphological and biophysical properties of neurons and neuronal networks that may have an effect on the generation of oscillations in a way that creates a non-zero OM activity.”

In the Discussion, 8th paragraph: “Critically, it means that low-frequency oscillations may carry relevant information about a high-frequency activity (here we use high-frequency and low-frequency as relative terms, not predetermined ranges), and thus both phenomena should be considered jointly.”

In the Discussion, the last paragraph: “This, in turn, provides further support for the idea that certain evoked responses and neuronal oscillations may share common neurophysiological mechanisms.”

The results are also presented in a way to somewhat over-emphasizes the subset of cases in which the baseline-high shift index is particularly high, first discussing, and only showing examples of high correlation, and not explicitly noting the number of cases that have a low correlation. This is despite the average baseline-shift of ~0.5 being a more modest finding, which is only noted a bit later in the results. It seems that many baseline-shift scores are close to zero is more consistent with baseline shift contributing / sometimes happening, rather being a clearly ubiquitous phenomenon. I think the results section on the empirical data could do better to acknowledge the variance in the findings. For example, instead of 3 selected and non-representative examples in Figure 3 (that are all quite similar), it may be useful to instead / also show examples from each group that show an example of the average baseline shift index – this would be more reflective of the pattern of results and help to visualize more common scenarios. Highlighting this variance could also surface a discussion of potential explanations of this variation. For example, it could be noted that this variance may be indicative of the extent to which other mechanisms may be at play.

We agree that the reader will be more interested in seeing different results. We edited fig.5 by adding two examples with near-zero baseline-shift indices. Additionally, we included permutation testing to obtain the number of BSI that are significantly different from zero.

Besides, please note that we provide comprehensive simulations (section Results/Effects of spatial synchronisation on the degree of alpha amplitude modulation and baseline shifts) with populational modelling showing one possibility how even when empirical BSI is zero (in the asynchronous network scenario, the alpha amplitude envelope is flat), evoked responses can nonetheless be generated through the baseline shift-mechanism. This is because there might be a considerable dissociation between the amplitude time course of neuronal sources and a corresponding low-frequency counterpart due to the phase cancellation of individual sources constituting a population of simulated neurons.

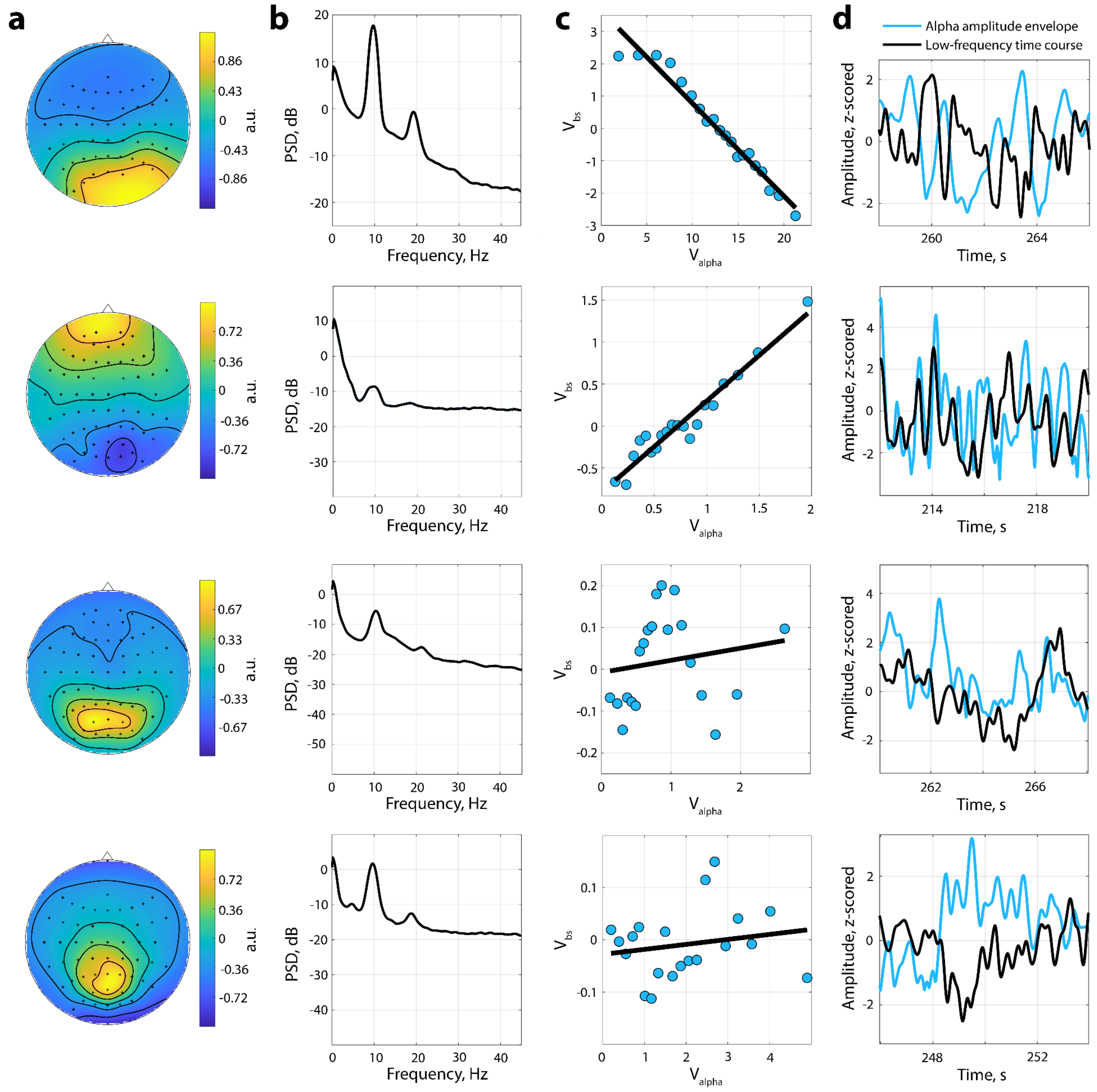

In the section Methods - Statistical analysis we added the following paragraph:

"To obtain a robust estimation of the significant BSIs, we applied permutation testing. Before binning, a low-frequency signal was cut at random points into 5 intervals (not exceeding 1.5 minutes), which subsequently were randomly shuffled. The

permutation distribution was built from computing BSI between the original alpha-filtered time course and shuffled low-frequency surrogate. The number of permutations was 500. The p-value was retrieved as the number of permuted BSIs that increased the original BSI divided by the total number of permutations. For the evaluation of significance, the sign of BSI was taken into account. The significance threshold was set to 0.05."

In section Results - Validating the baseline-shift mechanism on the large EEG data set we added the following lines:

"With permutation testing (see Methods), the total number of significant BSIs was 50% (53% for young participants, 48% for elderly participants). Critically, 93% of participants had at least one SSD-derived component with a BSI significantly different from zero."

In section Discussion:

"Based on permutation testing, 93% of participants had at least one example of alpha oscillations with BSI that was significantly different from zero, which in general suggests the ubiquity of the non-zero OM phenomenon."

Methodologically, the baseline shift is broadly a correlation between different frequency ranges in the data, computed from filtered traces. I wonder what the "null model" is for such a measure. For example, for white noise, this measure should, on average, be zero. However, neural data is not white noise, and the pink noise "background" of neural data implies that this measure would be expected to have some non-zero correlation, even absent of any oscillations. This seems to imply that one could see non-zero BSI values given the nature of the data, but absent of any non-zero mean oscillations. Has this measure been characterized in such cases to estimate the influence of colored noise signals on the data? Given the use of SSD, we can expect that the these components do contain oscillatory activity, but given the presence of 1/f activity (visible in Figure 5, for example), it would be useful to know the expected value of the BSI measure under an appropriate null model, to evaluate if and when the measured values are over and above this value. To the extent that this is not known, it may be important to at least acknowledge this potential issue. Conceptually, this issue is similar to investigations showing that, for example, that measures computed across different frequency ranges can be conflated by the overall 1/f activity of the data.
Related reference:
Donoghue, T., Dominguez, J., & Voytek, B. (2020). Electrophysiological frequency band ratio measures conflate periodic and aperiodic neural activity. Eneuro, 7(6).

We performed additional simulations and added the description to the Supplementary material.

The reference to supplementary material in the main text, the Results section:

"As BSI was previously introduced to quantify baseline shifts associated with alpha oscillations in empirical EEG/MEG data, before applying BSI to a large data set, we additionally juxtaposed its performance in simulations (see Supplementary material)."

The supplementary material:

"The quantification of baseline shifts via baseline-shift index (BSI; Nikulin et al., 2010) includes bandpass-filtered signal and low-frequency signal. It is now acknowledged and a widely researched matter that electrophysiological data is characterised by spectra with 1/f frequency distribution. Therefore, neuronal processes with a 1/f spectrum contribute to the signal in a broad frequency range and may thus affect the estimation of BSI. To test this, we performed additional simulations.

Methods

Firstly, we tested whether BSI would detect non-zero mean oscillations in a case when oscillations are absent. We simulated 900 time series of a 6-min-long signal consisting of either 1/f noise or a combination of 1/f noise and white noise (pink noise was simulated with python module colorednoise which is based on Timmer & Koenig, 1995). However, the actual null hypothesis for BSI is that alpha oscillations have a zero mean (see eq. 1). Therefore, secondly, we simulated 900 time series of a 6-min-long signal consisting of 1/f noise, and zero-mean or non-zero mean alpha oscillations. We simulated amplitude-varying alpha oscillation as a white noise filtered in the 8-12 Hz (Nikulin et al., 2011, Idaji et al., 2020). To construct non-zero mean oscillations, we summed oscillations with their rectified copy multiplied by a factor of 0.4. This situation scenario mimics exactly the eq. 1. For each time series, we computed BSI in the following way. From a composite time course, we obtained amplitude envelope (V_{alpha}) by applying the Hilbert transform to a band-pass filtered signal in the 8-12 Hz range and baseline shifts (V_{bs}) by low-pass filtering the signal at 3 Hz. After, V_{alpha} was divided into 20 percentile bins according to its magnitude. V_{bs} were sorted using the arrangement of the bins from V_{alpha}. The relation between V_{alpha} and V_{bs}, which is, in fact, a BSI, was estimated with the Pearson correlation coefficient (see also section Methods/The baseline-shift index). Each BSI was subjected to permutation testing to determine its significance (see section Methods/Statistical analysis).

Results

Signals that contained only noise did not show evidence for non-zero mean oscillations. For both 1/f noise or 1/f and white noise, the average BSI magnitude

was 0.37. However, the fraction of BSIs that were significantly different from zero based on permutation testing was at a chance level of 0.05. The null hypothesis that oscillations have a zero mean and the alternative hypothesis of non-zero mean oscillations were successfully evaluated with BSI as well. The average value of BSI was 0.36 for zero-mean oscillations and 0.85 for non-zero mean oscillations (at an average signal-to-noise ratio in the alpha band of 9 dB). The share of BSIs that were significantly different from zero based on permutation testing was 0.84 for non-zero mean oscillations. The share of non-zero BSIs for zero-mean oscillations was at the significance level."

Finally, I think the paper could benefit from some further discussions of the implications of the findings:
- In the introduction, the paper mentions some with results that are inconsistent with the baseline-shift mechanism (Fukuda et al, 2015 & Xia et al, 2020), and implies that this paper will address factors that might explain these conflicting results. However, this point is not revisited. How do the current results relate to such findings? It seems that this is consistent with baseline-shift explaining some of the variance of evoked potentials, but other mechanisms being at play, but the relation of this paper to prior reports with different findings is not revisited in the discussion, where it would be useful to do so.

We agree that we need to discuss the issue of discrepancies in the results. We expanded the paragraph in the discussion.

"Several previous studies observed evidence that was contrary to the hypothesis that alpha oscillations and evoked responses are manifestations of the same underlying process (Fukuda et al., 2015, Xia et al., 2020). However, based on our results, we believe that there are at least two potential reasons for their findings. Firstly, in our study, we applied spatial filtering with SSD to extract alpha oscillations with a high signal-to-noise ratio. Still, even spatially filtered components did not always demonstrate a non-zero mean property. We assume that this might have happened due to source mixing (both in the alpha band and in a low-frequency band) or individual anatomical differences in dipole locations. Consequently, it may be that Fukuda et al. in their study have observed an interplay of several alpha sources, from which only one was related to the evoked response. Secondly, the elderly and clinical populations may have alterations in oscillatory patterns, and Xia et al. recruited elderly participants and elderly patients with cognitive impairment. In our study, we found that the prominence of baseline shifts was on average lower in elderly participants. However, we observed that the correlation between the relative power of alpha and BSI magnitude was not strong. This indicates that there may be other influences that can explain a dissociation between the fluctuation of amplitude envelopes and corresponding baseline shifts. We hypothesized that one of such influential factors can be the strength of spatial synchronization and provided comprehensive simulations for this (see Results/Effects of spatial synchronisation on

the degree of alpha amplitude modulation and baseline shifts). Therefore, we believe that, in the study by Xia et al., the relation between alpha oscillations and ER was concealed due to changes in brain dynamics in the aged or/and diseased population."

- Based on the demonstrations (for example, Fig. 7), it seems that an implication of baseline shift is that evoked potentials themselves are not expected to be deflections around zero (the computed evoked responses of the simulations having non-zero mean). I presume the implication is that in real data, pre-processing steps such as demeaning or high-pass filtering may obfuscate this. Do these findings imply anything different that could be done in the analysis of evoked potentials to further explore the baseline-shift mechanism?

Indeed, further clarifications are required here. Firstly, one of the steps for the calculation of evoked responses is baseline correction. This is needed to eliminate possible differences due to technical drifts of an amplifier, electrode polarisation etc. Importantly, subtracting one value can not produce an evoked response per se. The demeaning would not change the shape of an evoked response, or the difference between evoked responses in different stimulus-conditions. Instead, according to the baseline-shift mechanism, evoked responses are produced by amplitude modulation of oscillations with a non-zero mean. However, in line with our simulations shown in fig. 7, it is conceivable that in empirical data non-zero values in baseline might be due to BSM. Secondly, it is correct that eliminating low frequencies might eventually eliminate baseline shifts that are associated with alpha oscillations. However, excessive high-pass filtering is not typically performed for evoked responses since otherwise, one would actually remove evoked responses. Consequently, the cut-off frequency for high-pass filters is usually below the frequency content of evoked responses. We added an explanation to the fig. 7 legend.

"The offset voltage of the evoked response in both cases is negative for two reasons: because we did not perform single-trial baseline correction, and because oscillations were simulated as having a negative mean. In case, when oscillations have a positive mean, non-corrected offset will be positive. After baseline correction, the offset voltage will be shifted to zero."

- Alpha oscillations are not continuously present across time and space. Relatedly this paper explicitly excludes patients that do not demonstrate clear alpha oscillations, which seems to be a potential issue give the goals of this analysis. Is the variability of oscillations a potential challenge to the baseline-shift proposal? What is the implication for subjects in which we do not observe clear oscillations?

We agree that the absence of oscillations might seemingly indicate that evoked responses can't be generated through the baseline-shift mechanism. In our sample, for young participants, we did observe a positive correlation between power in the

alpha band and the absolute value of BSI. It means that if oscillations are weak or even not present, the value of BSI would be closer to zero. However, it does not directly have implications for the baseline-shift mechanism itself. If we observe the lack of oscillations on the level of scalp data, we cannot be sure that oscillations are absent on the level of individual neurons. Possibly, oscillations are present but are rather desynchronised within the population, which affects the power. In fact, BSM does not take into consideration the phase of individual oscillators, and even when neurons are not perfectly synchronised within the population the baseline shift caused by modulation of the amplitude will arise. We have this reasoning in Discussion, 6th paragraph, p.15. Nonetheless, on the macroscopic level, the case when alpha oscillations are absent makes it harder to argue for BSM although it can't be excluded either. Having said that, in our sample, we eliminated only one participant's data exclusively based on the absence of a peak.
We also edited the paragraph in the Discussion in line with the above considerations:

"In our analysis of real EEG, we observed that the correlation between the relative power of alpha and BSI magnitude was generally positive, but not strong. Clearly, it means that the absence of observable macroscopic oscillations may obscure the evidence for BSM. However, it also indicates that there should be other factors, related or not related to oscillations, that can explain a discrepancy between the fluctuation of amplitude envelopes and corresponding baseline shifts. We hypothesised that one of the factors related to oscillations can be the strength of spatial synchronisation."

I understand that each of these points could be entire investigations themselves (and may be discussed in prior work that could be referred to), such that there may be minimal space to fully address these points, but given the potentially broad implications of this paper, I think it's worth trying to explore some of the points.
Figure notes:
- In Figure 5, in the power spectra, the steep drop off around 50 Hz is presumably due to the notch filter applied to the line noise. Since this drop off is irrelevant to the analyses, the spectra could be limited to a lower frequency to avoid visualizing this drop, as it's a bit distracting.

We agree with this remark. The figure had been edited. Please see one of the previous comments.

- In Figure 5, it might be useful to indicate which groups the examples come from.

According to one of the previous comments, we added examples of near-zero BSI. Additionally, we edited fig. 5 caption in the following way:

"Figure 5. Baseline shifts in rest EEG data. Examples of obtained SSD components topographies (Column a) and spectra (Column b). Column c. The association

between alpha amplitude envelope (Valpha) and low-frequency amplitude (Vbs) - baseline-shift index (BSI). BSI can be estimated as a slope of linear regression or as a correlation coefficient. For exemplar components, the first row - young group, BSI is negative, the second row, elderly group - BSI is positive, the third row - young group, BSI is close to zero, the fourth row - elderly group, BSI is close to zero. The non-linear relation in the third row may be a manifestation of an interplay between several alpha sources. Column d. Correspondence between alpha amplitude envelope and low-frequency signal. If BSI < 0, an increase in the alpha amplitude produces a baseline shift downwards. Contrary, if BSI > 0, an increase in the alpha amplitude produces a baseline shift upwards."

***Reviewer #2**: This paper examines the biophysical bases of non-zero mean alpha oscillations in electrophysiological data using realistic biophysical models and also a reanalysis of large EEG dataset. Results from simulations are quite interesting and worthy of publication. However, the paper is not as well organized. The introduction does not set up the questions, rationale and hypotheses being tested well. The reanalysis of large EEG data is not well justified. The final analysis of the role of spatial synchronization of alpha oscillators and how that could lead to differential empirical findings is not well described either. But the overall approach is noteworthy. Title is also a bit misleading and need context. What does it mean to be evident in empirical data?*

We are grateful for this comment. The main finding in our paper is that whenever we scrutinized a computational model or empirical EEG data, the evidence for non-zero mean in alpha oscillations was apparent. However, we agree that the current title requires improvement. We offer the following:

"Non-Zero Mean Alpha Oscillations Revealed with Computational Model and Empirical Data"

Abstract - The abstract is currently a bit too sparse. The ideas here extend also to iEEG and ECoG. A bit more needs to be said about the biophysical model and what parameter ranges were explored. What are participants in a model? What is the complementary neuronal-ensemble modeling? What discrepancy is being modeled and what is the explanation? What alternative hypothesis is ruled out?

We agree with considerations. We updated the abstract in line with this and the previous reviewer's comments.

"Ongoing oscillations and task-triggered evoked responses are two main types of neuronal activity obtained with diverse electrophysiological recordings (EEG/MEG/iEEG/LFP). Although typically studied separately, they might in fact be closely related. One possibility to unite them is to demonstrate that neuronal

oscillations have non-zero mean which predicts that stimulus- or task-triggered amplitude modulation of oscillations can contribute to the generation of evoked responses. We validated this mechanism using computational modelling and analysis of a large EEG data set. With a biophysical model, we indeed demonstrated that intracellular currents in the neuron are asymmetric and, consequently, the mean of alpha oscillations is non-zero. To understand the effect that neuronal currents exert on oscillatory mean, we varied several biophysical and morphological properties of neurons in the network, such as voltage-gated channel densities, length of dendrites, and intensity of incoming stimuli. For a very large range of model parameters, we observed evidence for non-zero mean of oscillations. Complimentary, we analyzed empirical rest EEG recordings of 90 participants (50 young, 40 elderly) and, with spatio-spectral decomposition, detected at least one spatially-filtred oscillatory component of non-zero mean alpha oscillations in 93% of participants. In order to explain a complex relationship between the dynamics of amplitude-envelope and corresponding baseline shifts, we performed additional simulations with simple oscillators coupled with different time delays. We demonstrated that the extent of spatial synchronization may obscure macroscopic estimation of alpha rhythm modulation while leaving baseline shifts unchanged. Overall, our results predict that amplitude modulation of neural oscillations should at least partially explain the generation of evoked responses. Therefore, inference about changes in evoked responses with respect to cognitive conditions, age or neuropathologies should be constructed while taking into account oscillatory neuronal dynamics.”

Introduction - Distinction between phase-locked vs non-phase-locked task-induced neural oscillation should be made clear upfront.

We agree. We added a sentence in the second paragraph of the introduction.

“Note that the BSM scenario supposes non-phase-locked oscillations with non-zero OM; when averaging over trials, opposite phases of oscillations cancel out and ER appears in the cumulative signal.”

It is very strange to say "...there is no clear cut evidence in strong support of either of mechanisms." when the authors have cited so many papers for each type of mechanisms for the evoked response.

We agree that this sentence was somewhat misleading and did not reflect what we wanted to point out. We rewrote this part of the paragraph:

“There is an ongoing debate in the literature on whether auditory (...), visual (...), and somatosensory (...) evoked responses can be generated through the additive, phase reset mechanism, or BSM, and currently, the evidence is inconclusive for some evoked responses and completely absent for others. It should be noted that these

mechanisms are not assumed to be mutually exclusive and may co-exist. Moreover, they may manifest at different latencies of evoked-response generation and in different conditions. The third mechanism - BSM - was introduced relatively recently and so far was underexplored in studies and computational models. In the current study, we reinforced the theory behind BSM."

Seems redundant to say "The third mechanism - BSM - is based on two prerequisites: 1) sensory stimuli or movements should modulate the amplitude of ongoing oscillations (these ndings being conrmed in almost all EEG/MEG studies on oscillations); 2) neuronal oscillations have a non-zero OM. ..." when this point has just been stated in the previous paragraph. The subsequent first two predictions are also a repetition of the same points. Isnt the third prediction mentioned in this paragraph just a restatement of the first one that - if evoked responses are a result of post-stimulus induced amplitude modulations their timing and spatial distribution would be similar?

Due to the relative unfamiliarity of the baseline-shift mechanism in the community, we believe that repeating prerequisites and predictions are helpful for its proper understanding. However, we agree that exact reiterations might be redundant, especially in the adjunct paragraphs. Therefore, we removed the prerequisites from this paragraph.

A bit more need to be said about the two studies that did not find evidence for such correlation. Actually, it is unclear that is what was found in the Fukuda et al. study. They suggested that both amplitude modulation and evoked responses independently contribute to working memory effects rather than being a unitary process but could that be something unique to memory encoding?

We are thankful for this comment. The matter of discrepancies is indeed insufficiently described. We expanded the paragraph.

"However, several other studies observed little or no correlation (Fukuda et al., 2015, Xia et al., 2020). Fukuda et al. found counter evidence for alpha modulation and evoked response relatedness in the visual working memory task. The main finding was that the strength of alpha amplitude and amplitude of evoked response were not correlated between different memory array sizes. Xia et al. examined evoked response P600 and suppression of alpha activity in the verbal memory task in healthy elderly participants and in patients with mild cognitive impairment and Alzheimer's disease. The study showed two phenomena to be distinct based on the fact that they lacked correlation on a single trial level and acted as complementary metrics in multiple regression while predicting California Verbal Learning Test scores. In the current study, we considered factors that might have caused conflicting results. We assumed that, possibly, not every alpha source presents detectable baseline shifts on the level of EEG/MEG, and that the estimation of baseline shifts

may be complicated in the elderly or clinical population (for instance, due to the low power of oscillations Rossini et al., 2007).”

"On a membrane level ... placed asymmetrically on a neuron ..." What does asymmetry refer to here? On a cellular level how does orientation contribute to asymmetry? Finally on the exogenous inputs level, again it is unclear what is symmetric and what is being referred to as asymmetry here. Overall this paragraph needs to be made clear. The last two sentences are also confusion because there is a suggestion that some of this is already known, if so what is new that is being investigated here.

We have expanded the paragraph in the following way:

“The intuition about oscillations having non-zero OM stems from asymmetrical morphological and biophysical properties of neurons and neuronal networks that may have an effect on the generation of oscillations in a way that creates a non-zero OM activity. These asymmetries emerge on several scales: a membrane, a cell, and external inputs. On the membrane level, inward and outward currents flow through synapses and voltage- and ligand-activated channels that are placed asymmetrically along the neuron (....). The placement of synapses over the dendrites of one cell is biased in a way that excitatory synapses are clustered on spines of apical and basal dendrites, and inhibitory synapses are mostly located on dendritic shafts, soma and axon initial segment (Bannister, 2005, Boivin & Nedivi, 2018). In addition to synapses, charges flow through voltage- and ligand-gated channels that are scattered along the membrane across all parts of dendrites and soma (Bekkers, 2000, Benhassine & Berger, 2005). On the cellular level, the contribution of various dendrites to a current dipole is not equal and depends on the orientation of a process with respect to the longitudinal axis of the apical dendrite (...). For instance, oblique dendrites, which are oriented in parallel to the cortical surface and transversely to the main axis of a neuron, have little effect on the net dipole. As for the basal dendrites, they are oriented in different directions (schematic representation on Fig. 1), meaning the contribution of primary currents in basal dendrites is scaled to some degree (Neymotin et al., 2020). On the exogenous inputs level, proximal and distal drives arrive at different layers of the neocortex, thus creating an asymmetrical redistribution of charges (...). In particular, feedforward thalamocortical drive arrives predominantly to basal dendrites of pyramidal neurons, whereas feedback connections - to apical dendrites. The strength of thalamocortical and corticocortical connections may change irrespectively from each other, thus creating asymmetries in currents. Overall, based on theoretical assumptions about neuronal currents, it is highly unlikely that currents flowing towards soma will have the same magnitude as outward dendritic currents (...). However, as previous research on BSM was focused on EEG/MEG findings, we checked the aforementioned theoretical statements with a computational model. We supposed that the change in parameters of the model

would bring about the change in cellular currents. Subsequently, when currents become more or less balanced, the mean of oscillations would change."

Additionally, we edited a sentence in the Results/HNN section:

"Owing to the fact that electric and magnetic fields are proportional to primary currents (Ilmoniemi & Sarvas, 2019), to simulate the amplitude of a signal that is typically observed in macroscopic recordings, oscillations of 200 pyramidal neurons are then multiplied by 300,000 (Neymotin et al., 2020) so that 60,000,000 pyramidal neurons give rise to the signal."

The paragraph starting with "Despite that ..." refers to previous studies but has no citation. The point of this paragraph is unclear.

We edited the paragraph in the following way:

"Despite that, there is no strict necessity to measure OM directly. If neuronal oscillations have a non-zero OM, any modulation of oscillations' amplitude affects the mean as well, thus leading to a baseline shift in lower frequency (Nikulin et al., 2007, Nikulin et al., 2010). Consequently, in the macroscopic recordings, there would be an association between low-frequency time course and the amplitude envelope of ongoing oscillations (see eq. 1 in the Methods section). This association is present in both stimulus-based and rest-state data because alpha rhythm modulation is present during both active and resting states. Therefore, for BSM, amplitude modulation bears a significant role both for the observation of evoked responses and for the empirical estimation of OM. However, the prominence of amplitude modulation may change. Therefore, as previous research sampled data exclusively from young adults and only during either eyes-closed or eyes-open state (Nikulin et al., 2007, Mazaheri and Jensen, 2008, Iemi et al., 2019), and the sample size was relatively small, we validated BSM on the bigger data set with both eyes-closed and eyes-open conditions and with two age groups. We expected to see a manifestation of baseline shifts in the majority of participants but also hypothesised that elderly participants may deviate from young participants in the manifestation of baseline shifts. A decrease of spectral alpha power is a prominent feature of the ageing brain (Rossini et al., 2007), and this issue may obscure the identification of a correspondence between alpha envelope and low-frequency baseline shifts."

There needs to be a clearer motivation for the use of the HNN. What exactly is the validation in a larger dataset? What is new here? What is actually being done here? The final goal of the study is quite vague. What are some hypotheses that are being tested here? Overall, the goals and hypotheses need to be more clearly laid out here.

We agree with the necessity to sharpen the aims and goals better. We have rewritten three paragraphs in line with this and previous comments. The edited paragraphs are above.

Results - the color scheme in figure 1b needs to be explained more clearly. What is the main focus of this figure? A bit unclear if the frequency of oscillations generated in figure 2 are due to the frequency of the proximal or distal input drive?

We have added the extended description to fig. 1b.

“b. A schematic illustration of the effect that incoming inputs exert on the dendritic currents. The proximal drive is a simulation of thalamic activity coming to the granular layer of the cortex, which is later relayed to layer II/III and layer V. Distal drive is excitatory input from non-lemniscal thalamic sources and/or other cortical areas (see Methods). Proximal connections terminate on the basal dendrites of pyramidal neurons, while distal input is reaching apical dendrites. Therefore, currents that are generated in the neuron as a response to proximal stimulation flow predominantly in the upward direction (with respect to the surface of the cortex), and distal drive creates downward currents (...). Spontaneous alpha rhythm emerges when the delay between proximal and distal inputs is 50 ms, and both are delivered to the network with a frequency of 10 Hz (Ziegler et al., 2010, Kerr, Sacchet, Lazar, Moore, & Jones, 2013, Neymotin et al., 2020). However, simultaneous presence of both proximal and distal inputs is not essential for the emergence of the alpha rhythm (see Fig. 3). The current figure demonstrates the most biologically plausible layout of inputs’ allocation.”

WIth the intro suggesting membrane, cellular and network level properties as contributors to non-zero OM it may make more sense to present the results in a parallel structure.

We agree with the advantage of a parallel structure. We swapped fig.2 and fig.3 and the corresponding paragraphs of the text.

It seems like there is no zero-mean in the model across a range of parameters. Are they any conditions where they will be zero-mean? There seems to be a clue in figure 3B but nowhere else.

We acknowledge that for a limited range of model parameters one can create a zero-mean oscillation. We also believe that the neuronal network generates ever-changing patterns. One cannot set the strength of thalamic input to a particular value and assume that it would remain the same. With the model, we aimed to show that dendritic currents are unbalanced/asymmetric and this asymmetry leads to non-zero mean oscillations. In the 5th paragraph of the Introduction, we added explicit statement: “We supposed that the change in parameters of the model would

bring about the change in cellular currents.“; and added a statement in the 3rd paragraph of Discussion: “From our simulations, it also follows that fluctuations in currents may on rare occasions lead to close-to-zero mean of oscillations. ”.

The varying axes in figure 3 is also confusing but interesting. Is the DC levels expected to be much smaller than observed oscillatory amplitudes? That seems to be the case in some of the examples but not in all. It may help to look at the relative alpha power and the OM together.

We are thankful for this observation. The mean of oscillations is indeed somewhat smaller than the spread of amplitude of oscillations. However, in the HNN model, the ongoing change in power is not introduced yet. In empirical electrophysiological recordings, the power of oscillations directly depends on the number of coherently oscillating neurons. Under this assumption, we changed the network-scaling parameter which reflects the number of neurons in the model. In this case, the mean of oscillations increases linearly with the number of oscillating neurons (a double increase in the number of neurons corresponds to a double increase in the mean). However, we would like to avoid making exact claims about the size of the mean or its sign in real neuronal ensembles based solely on simulations. Yet, we add the following sentence in the Discussion: “Although the mean of oscillations is relatively small compared to the amplitude of oscillations, it can still be amplified by the number of synchronously active neurons.” Importantly, amplitude fluctuations of oscillations can be largely cancelled, yet baseline shifts would remain since they are insensitive to the phase alignments.

A bit more needs to be said about how figure 4d is computed from the other parts of this figure. I think a figure showing how the BSI is computed will be helpful in this figure. The meaning of the BSI slope should also be better explained. "The sign of BSI is positive which is consistent with OM of simulated oscillation. BSI " What does this mean?

We expanded the caption of the figure.

“Alpha modulation from real EEG is superimposed on time series simulated with default settings in HNN. a. Modulated oscillations. For this case, OM is positive. b. A spectrum of the modulated signal. c. Correspondence between alpha amplitude envelope and simulated time course filtered in a low-frequency range. Since OM is positive, an increase in alpha amplitude is associated with a baseline shift upwards. d. Baseline-shift index (BSI; …) applied to a simulated and modulated signal. BSI expresses a relation between low-frequency signal and the ongoing rhythm envelope (in the current study, alpha oscillations and associated baseline shifts in the 0.1-3 Hz range). Values of amplitude envelope and low-frequency signal are binned in 20 bins and the relation is estimated as a slope of linear regression or as the Pearson coefficient. The sign of BSI reflects the sign of the underlying OM. For this case, the

sign of BSI is positive which is consistent with OM of simulated oscillations. Here, BSI computed as a Pearson coefficient is 0.98."

Figure 5 analysis is not well motivated. There is no figure on the correlations between BSI and low-frequency etc.

We edited fig.5 in line with this comment and the comments of the first reviewer, as well as made additions to the main text.

"For the top two examples, the BSI or the correlation between alpha amplitude envelope and low-frequency amplitude is more than 0.9 (BSI was computed as the Pearson correlation coefficient). For the bottom two examples, the BSI is close to zero. Fig. 5 demonstrates how some alpha oscillations have a straight linear relation between their amplitude and amplitude of a low-frequency signal, whereas others do not show this kind of relation."

Additionally, we substituted the table on the correlation with the following figure.

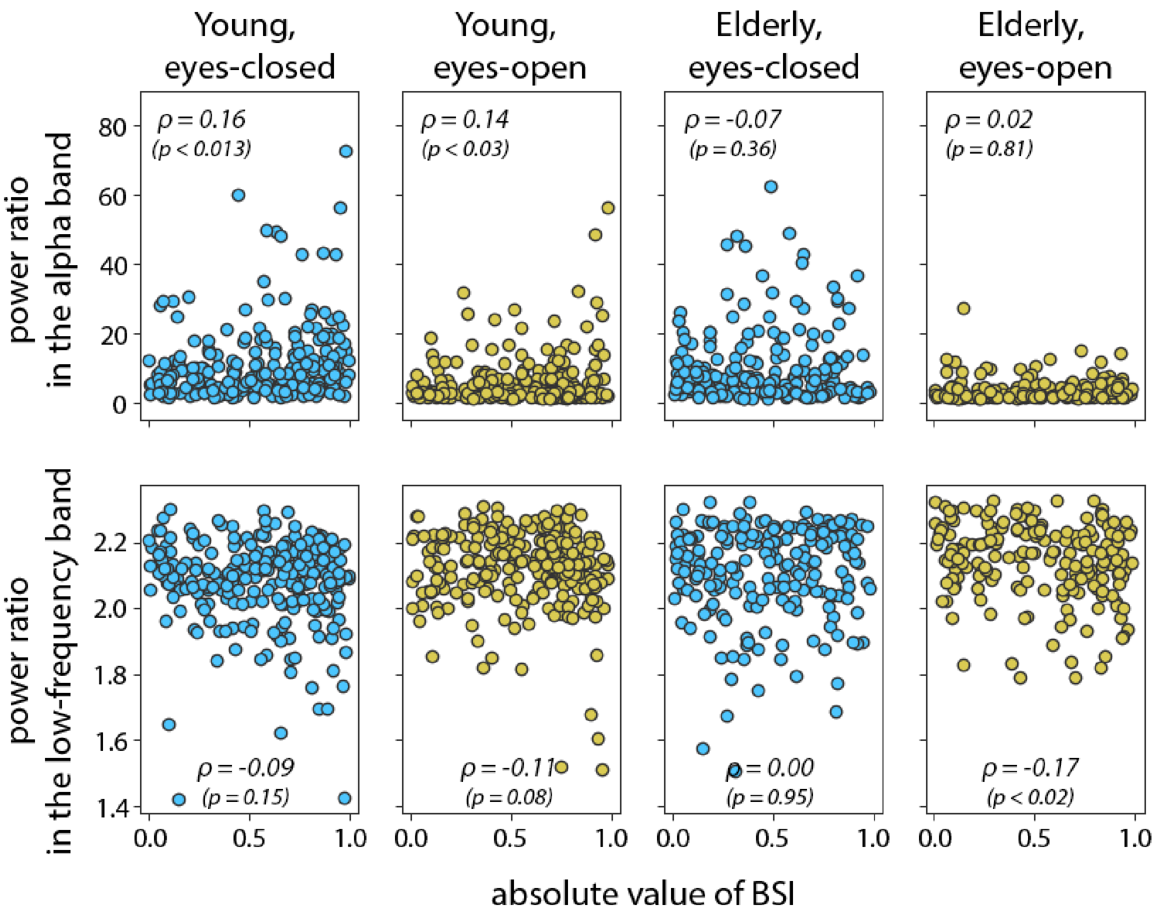

"Figure 6. Correlation between the absolute value of BSI versus power ratio in the alpha band and power ratio in the low-frequency band for pairs of age groups and

conditions. The correlation was computed with the Pearson correlation coefficient. The p-value is presented in the brackets."

What is the goal of the ANOVA described in page 8? What hypotheses are actually being tested here and why?

According to previous comments, we added the hypothesis in the Introduction. The ANOVA was motivated by our expectations to see the difference between young and old participants. We also wanted to test whether the power in the alpha band could solely account for this difference. We appended an extension to the ANOVA paragraph in the Results section.

"We observed differences in BSI absolute values in young and old participants, in agreement with our hypothesis. Absolute BSI values had a tendency to be larger in the young group (0.564 ± 0.012 in the young group, 0.494 ± 0.015 in the elderly group when averaged across conditions ± standard error of the mean). Figure 6 shows the corresponding data. To test whether the difference in absolute BSIs was significant between the groups and conditions we applied ANOVA. Additionally, we expected that the power of oscillations may explain the difference between groups. Therefore, we extended the ANOVA model with covariate variables such as power ratio in the alpha band and the power ratio in the low-frequency band that are known to differ in age groups and eyes-open and eyes-closed conditions and might have an effect on BSI."

The transition from simulations to real data is abrupt.

We expanded the first paragraph of the EEG results section.

"Simulated data provide robust and stable oscillations that possess little variation in amplitude and frequency. However, in real data, oscillations tend to occur with highly varying amplitude including periods of no oscillations (Donoghue, Schaworonkow, & Voytek, 2021). That being the case, we investigated the presence of non-zero mean alpha oscillations in a large data set containing resting-state EEG data. We utilized resting-state data because even during rest, the amplitude of alpha oscillations significantly fluctuates over time which in turn should be associated with corresponding baseline shifts. To expand the assessment, we analyzed data from 90 participants (50 young, range 20–35 years, and 40 elderly, range 60–80 years, participants) with both eyes-closed and eyes-open sessions."

The section on effects of spatial synchronization on alpha amplitude modulation and baseline shifts is also quite poorly motivated. Figure 7 is poorly explained.

We augmented the beginning of the section with additional rationale. We also edited the fig. 7 capture.

“Contrary to our hypothesis, EEG findings indicate that power only weakly correlates with the magnitude of BSI. We observed a significant correlation of BSI and power in the alpha band only for young, but not for elderly participants. In addition to power, for the older population, a decrease in the synchronization in the alpha band was reported before (Vysata et al., 2014). Therefore, we anticipated that spatial synchronization among neurons generating oscillations may affect the amplitude of alpha rhythm, and consequently affect the estimation of BSI. Using population modelling (Schaworonkow and Nikulin, 2019), we show that the degree of spatial synchronization has indeed a strong impact on the amplitude of macroscopic alpha oscillations while not having a considerable influence on the generation of the baseline shifts.

Figure 7. Synchronous and asynchronous networks. The synchronous network contained oscillators with small phase delays. Whereas, the asynchronous network had large phase lags between the individual oscillators. At the beginning of the simulation, phases were sampled from von Mises distribution with different concentration settings corresponding to different degrees of within-population synchronization. κ is the concentration parameter that tunes the spread of phases at the beginning of an epoch. The central value of the distribution of starting phases for the displayed epoch is marked by the black arrow. Amplitude modulation was modelled as inverted Gaussian with varying widths of the left and right flanks. The network contained 30000 oscillators, the simulation was repeated 100 times to mimic the stimulus-based paradigm. A synchronous network displayed a significantly more pronounced response of the alpha rhythm envelope. For an asynchronous network, the amplitude envelope remained monotonously flat. However, in both cases, individual oscillators underwent the same amplitude modulation.”

Much of the discussion seems to be a restatement of many of the results and the broader implications are somewhat missing, as are alternative interpretations and confounds from prior studies etc.

In line with previous comments, we added to the Discussion section our opinion on Fukuda et al, 2015 and Xia et al, 2020 (see earlier). Additionally, we supplemented the Discussion with a limitation paragraph (below).

“The current study has several limitations that could be potentially addressed in future studies. In the analysis of EEG data, we used spatial filtering with SSD which helped to retrieve alpha oscillations with a high signal-to-noise ratio. Yet these components can still reflect a mixture of oscillations with varying directions of baseline shifts. Moreover, we did not set any specific hypothesis about the location or function of the alpha rhythm with a non-zero mean. Instead, we explored all

available SSD components containing alpha oscillations and verified their agreement with the baseline-shift mechanism. On the contrary, if the research question involves a certain evoked response such as P300, source reconstruction would be necessary. If BSM is to be tested for a particular evoked response, one should examine oscillations that may be associated with this response in the source space. Furthermore, the question of frequency tuning remains unanswered. For the computation of BSI, we bandpass filtered the broadband data around the individual alpha peak. However, for low frequency, we used the same predetermined range from 0.1 Hz to 3 Hz. Possibly, the range of alpha modulation may differ in participants and even within one participant for different alpha sources. In the light of recent studies that show how alpha frequency ranges may differ across the population, even with speculations that for some participants alpha oscillations may lie outside the typical alpha range (Haegens et al., 2014), we assume that more careful frequency tuning may be beneficial for the detection of baseline shifts."
